# Rapid production of SARS-CoV-2 receptor binding domain (RBD) and spike specific monoclonal antibody CR3022 in *Nicotiana benthamiana*

**DOI:** 10.1038/s41598-020-74904-1

**Published:** 2020-10-19

**Authors:** Kaewta Rattanapisit, Balamurugan Shanmugaraj, Suwimon Manopwisedjaroen, Priyo Budi Purwono, Konlavat Siriwattananon, Narach Khorattanakulchai, Oranicha Hanittinan, Wanuttha Boonyayothin, Arunee Thitithanyanont, Duncan R. Smith, Waranyoo Phoolcharoen

**Affiliations:** 1 Research Unit for Plant-Produced Pharmaceuticals, Chulalongkorn University, Bangkok, Thailand; 2 Department of Pharmacognosy and Pharmaceutical Botany, Faculty of Pharmaceutical Sciences, Chulalongkorn University, Bangkok, Thailand; 3 Department of Microbiology, Faculty of Science, Mahidol University, Bangkok, Thailand; 4 Department of Microbiology, Faculty of Medicine, Universitas Airlangga, Surabaya, Indonesia; 5 Institute of Molecular Biosciences, Mahidol University, Salaya, Nakhon Pathom Thailand

**Keywords:** Biotechnology, Immunology, Molecular biology, Plant sciences

## Abstract

Severe acute respiratory syndrome coronavirus-2 (SARS-CoV-2) is responsible for the ongoing global outbreak of coronavirus disease (COVID-19) which is a significant threat to global public health. The rapid spread of COVID-19 necessitates the development of cost-effective technology platforms for the production of vaccines, drugs, and protein reagents for appropriate disease diagnosis and treatment. In this study, we explored the possibility of producing the receptor binding domain (RBD) of SARS-CoV-2 and an anti-SARS-CoV monoclonal antibody (mAb) CR3022 in *Nicotiana benthamiana.* Both RBD and mAb CR3022 were transiently produced with the highest expression level of 8 μg/g and 130 μg/g leaf fresh weight respectively at 3 days post-infiltration. The plant-produced RBD exhibited specific binding to the SARS-CoV-2 receptor, angiotensin-converting enzyme 2 (ACE2). Furthermore, the plant-produced mAb CR3022 binds to SARS-CoV-2, but fails to neutralize the virus in vitro. This is the first report showing the production of anti-SARS-CoV-2 RBD and mAb CR3022 in plants. Overall these findings provide a proof-of-concept for using plants as an expression system for the production of SARS-CoV-2 antigens and antibodies or similar other diagnostic reagents against SARS-CoV-2 rapidly, especially during epidemic or pandemic situation.

## Introduction

An outbreak of coronavirus disease 2019 (COVID-19) was reported very recently in late December 2019 in one of the largest cities in China, Wuhan, Hubei province which was later confirmed to be caused by the betacoronavirus severe acute respiratory syndrome coronavirus 2 (SARS-CoV-2; formerly known as 2019-nCoV). This zoonotic virus is believed to have originated from animals and was transmitted to humans by an animal-to-human spillover event linked with a local seafood and animal market in Wuhan. The infection spread in mainland China rapidly and subsequently expanded to multiple countries mainly through human movement. Many confirmed cases of COVID-19 have been reported worldwide, with more than 33 million infected cases and more than 1 million deaths altogether in 6 continents as of September 2020, with a variable mortality rate^1–4^.

Outbreaks of other similar coronaviruses including severe acute respiratory syndrome coronavirus (SARS-CoV) and Middle East respiratory syndrome coronavirus (MERS-CoV) in 2003 and 2012 respectively also caused severe and often fatal illness in humans. Although the pathogenicity of SARS-CoV-2 might be similar or higher as compared to SARS-CoV and MERS-CoV, it is inappropriate to predict the pathogenicity of the virus at this stage. Human-to-human transmission has been reported through respiratory droplets or through close contact with an infected person, which has caused widespread fear and concern over this disease^5–7^. Currently, SARS-CoV-2 has emerged as a global public health concern, with many people being infected around the world, and the World Health Organization (WHO) has declared this coronavirus outbreak as a Public Health Emergency of International Concern and characterized COVID-19 as a pandemic^8^. To date, there is no specific treatment or vaccine available to treat COVID-19 infections, and research in these areas is currently in progress. Hence, there is an urgent need to develop rapid diagnostic methods, vaccines and therapeutics to tackle the COVID-19 outbreak and control the virus spread. Currently, the global priority is to improve the availability of diagnostic services especially to people living in developing and under-developed countries, thereby reducing the massive spread of the virus and the mortality associated with it. Hence, the continued spread of SARS-CoV-2 in many countries demands the development of cost-effective rapid COVID-19 diagnostics for surveillance. The sudden increase in the demand for diagnostic protein reagents requires a flexible protein production platform that can rapidly produce the reagents affordably to address the global public health crisis.

Over the past two decades, biopharmaceuticals have been produced in a number of different expression systems such as yeast, mammalian cells, and plants, but currently most of the commercially available recombinant vaccines or biopharmaceuticals are produced in mammalian or microbial cell cultures. One of the major issues with the biopharmaceuticals produced in mammalian system is the requirement of initial capital investment and the high production costs associated with it^9,10^. While each expression system has its own advantages, they have all proven to possess distinct shortcomings, and the limitations of each expression system led to development of alternative production platforms that could significantly reduce the production costs. Recently, plants have emerged as an effective recombinant protein production platform, as they offer many advantages over conventional platforms such as economy, flexibility, rapid scalability and safety. Previous reports have demonstrated the potential of plant transient expression systems for the rapid production of proteins of pharmaceutical importance^11–18^.

Several groups have characterized potent antibodies targeting the coronavirus spike protein that effectively neutralize SARS-CoV in vitro and in vivo^19–27^. Based on the recent report by Tian and colleagues (2020), neutralizing antibody CR3022 obtained from a convalescent SARS-CoV infected patient was reported to potently bind with receptor binding domain (RBD) of the SARS-CoV-2 spike protein^26^, and therefore represents an important candidate mAb with potential as a therapeutic molecule, alone or in combination with other potential candidates for treating COVID-19. Given the ability of plants to assemble functional antibodies and antigens, the ease and speed of functional protein production, we employed a plant expression system for the rapid production of SARS-CoV-2 RBD and mAb CR3022, which could be applied as a vaccine, therapeutic, or diagnostic reagent for COVID-19.

The present study demonstrates the rapid production of the RBD of SARS-CoV-2 and mAb CR3022 in *Nicotiana benthamiana* using a transient expression system. The plant-produced RBD showed specific binding to the receptor of SARS-CoV-2, angiotensin-converting enzyme 2 (ACE2), confirming its structural integrity. Further, the plant-produced mAb CR3022 exhibited binding to SARS-CoV-2, but it failed to neutralize the virus in vitro. Overall, this study provides a proof-of-principle for the rapid production of SARS-CoV-2 antigens or monoclonal antibodies in a plant expression system in order to produce diagnostic reagents, vaccines and therapeutics which are highly needed during infectious disease outbreaks.

## Results

### Expression and purification of RBD of SARS-CoV-2 in *N. benthamiana*

**Figure 1:**
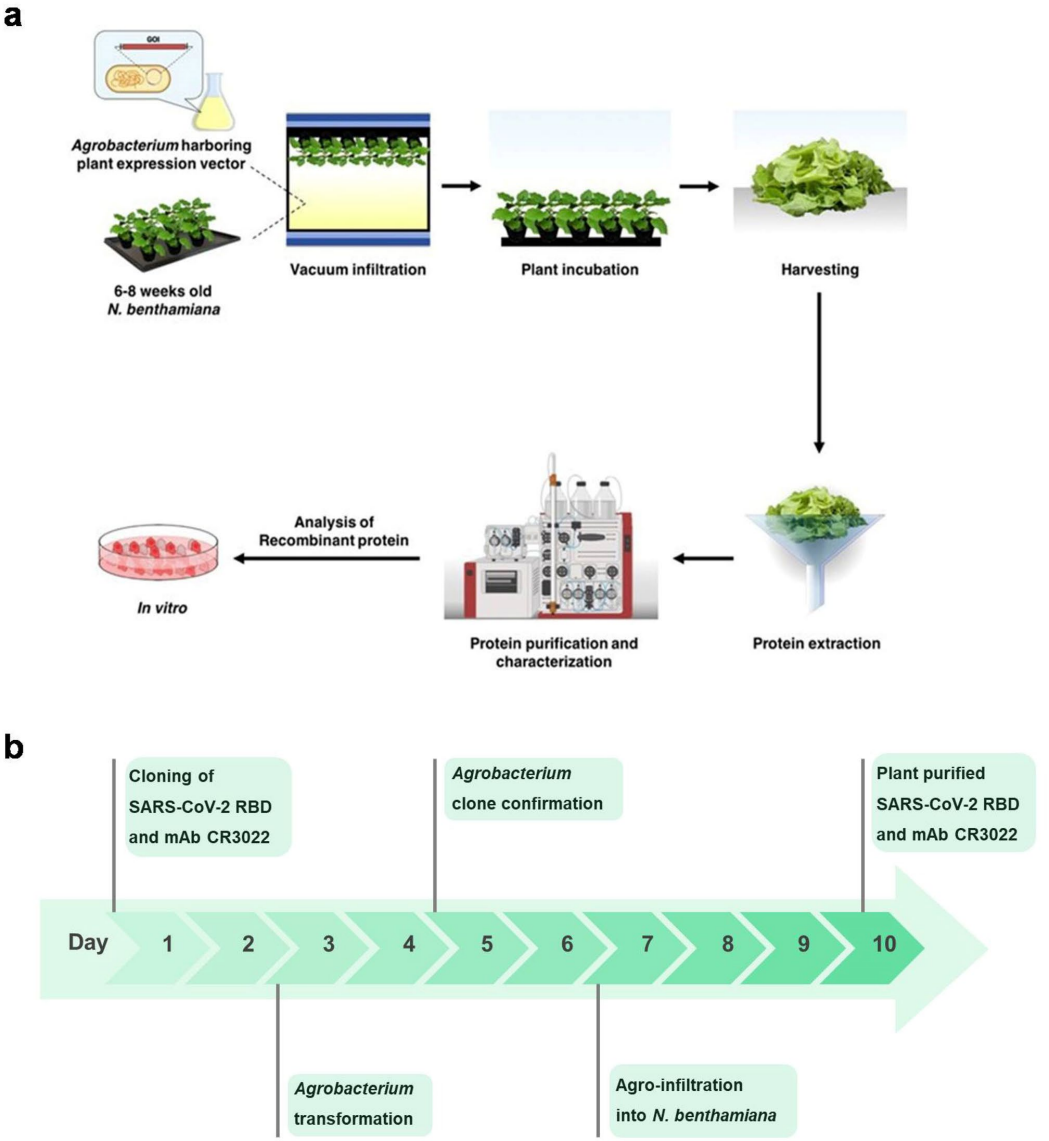
Schematic representation (**a**) and timeline (**b**) for the production of recombinant SARS-CoV-2 RBD and mAb CR3022 in plants by transient gene expression.

**Figure 2:**
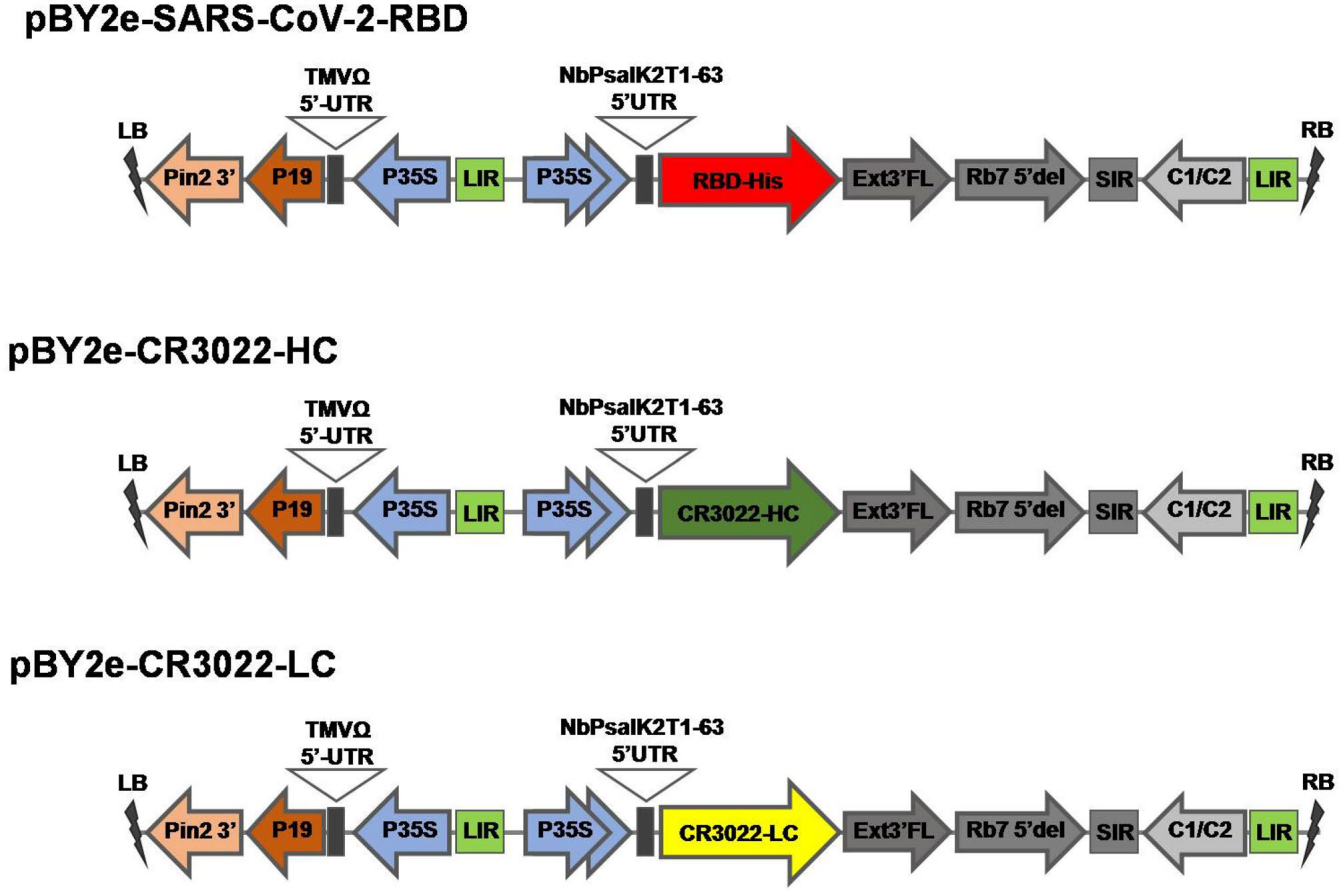
Schematic diagram of the T-DNA region of the plant expression vector used in the present study. RB and LB, the right and left borders of the T-DNA region transferred by *Agrobacterium* into plant cells; P35S: Cauliflower Mosaic Virus (CaMV) 35S promoter, NbPsalK2T1-63 5′UTR: 5′ untranslated region, RBD: SARS-CoV-2 RBD, CR3022 HC: heavy chain of CR3022 antibody, CR3022 LC: light chain of CR3022 antibody, Ext3′FL: 3′ region of tobacco extension gene, Rb7 5′ del: tobacco RB7 promoter, SIR: short intergenic region of BeYDV, LIR: long intergenic region of BeYDV, C2/C1: Bean Yellow Dwarf Virus (BeYDV) ORFs C1 and C2 encoding for replication initiation protein (Rep) and RepA, TMVΩ 5′-UTR: 5′ untranslated region of tobacco mosaic virus Ω, P19: the RNA silencing suppressor from tomato bushy stunt virus; PinII 3′: the terminator from potato proteinase inhibitor II gene.

**Figure 3:**
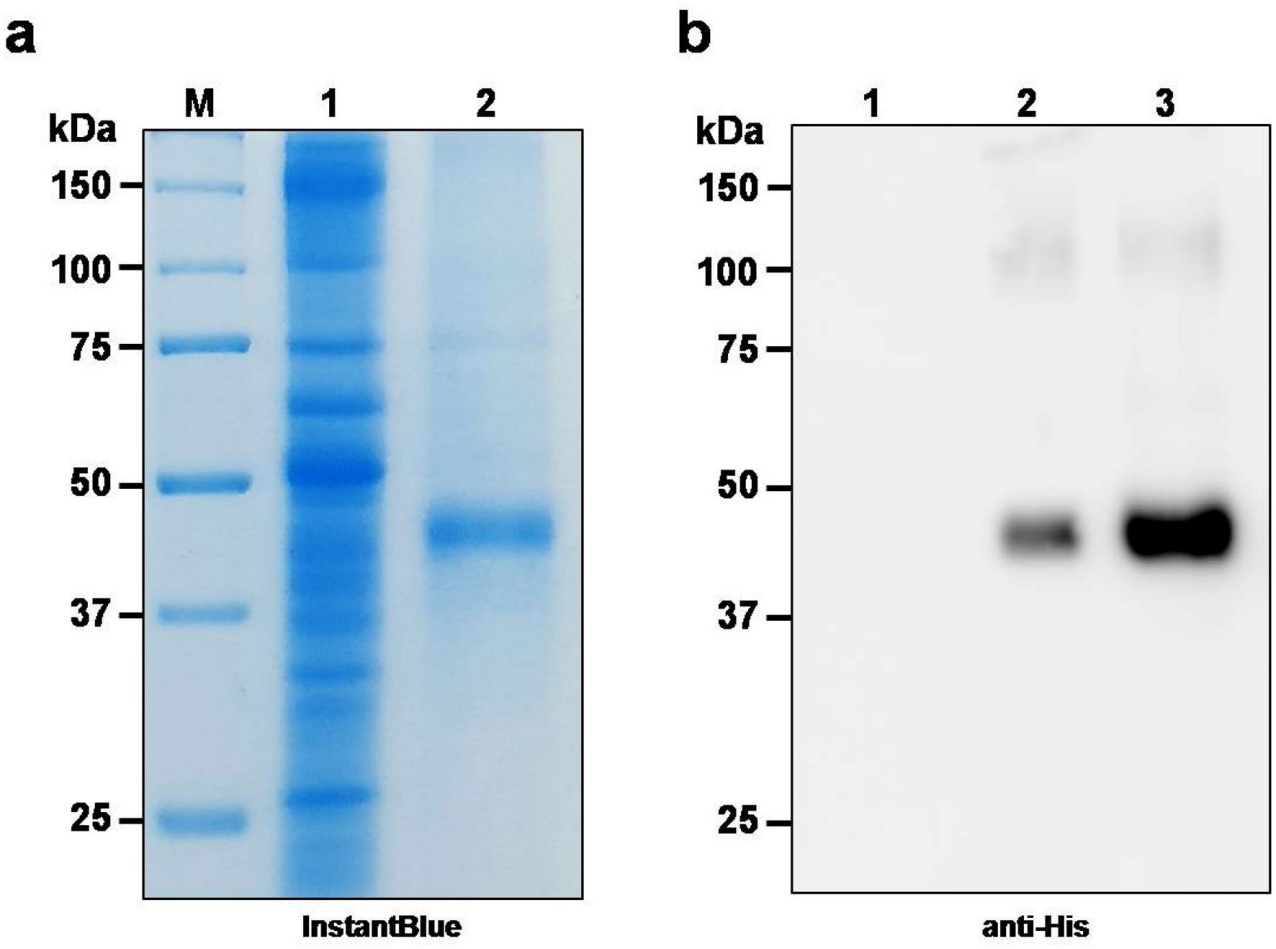
SDS-PAGE and western blot analysis of RBD protein of SARS-CoV-2 produced in *N*. *benthamiana*. The crude proteins were extracted from plants, and the RBD protein was purified, analyzed on SDS-PAGE gels and visualized with InstantBlue™ (**a**). Lane M: protein ladder; Lane 1: total soluble protein of *N. benthamiana* agroinfiltrated with pBY2e-SARS-CoV-2 RBD; Lane 2: purified SARS-CoV-2 RBD. For western blot analysis, proteins on the blot were probed with a rabbit anti-his antibody conjugated with HRP (**b**). Lane 1: crude extract from non-infiltrated *N*. *benthamiana*; Lane 2: total soluble protein of *N. benthamiana* agroinfiltrated with pBY2e-SARS-CoV-2 RBD; Lane 3: purified SARS-CoV-2-RBD.

The schematic representation and the timeline for recombinant protein production in plant system is given in Fig. 1a,b respectively. The codon-optimized RBD of SARS-CoV-2 was cloned into the geminiviral plant expression vector pBY2e (Fig. 2) and agroinfiltrated into *N. benthamiana*. The infiltrated leaves were harvested 3 days post-infiltration and the recombinant RBD protein was purified by Ni affinity chromatography. The expression and purification of the recombinant RBD protein were evaluated by SDS-PAGE and western blot analysis. The RBD protein was observed at the expected molecular weight of approximately 38 kDa in SDS-PAGE (Fig. 3a, Lane 2) and western blot (Fig. 3b, Lane 2 and 3). Further a faint band was detected in the western blot at about 120 kDa, which could be protein trimer (Fig. 3b). The expression level of the RBD of SARS-CoV-2 was estimated to be 8 μg per gram leaf fresh weight.

### Binding of plant-produced RBD to ACE2, the receptor of SARS-CoV-2

**Figure 4:**
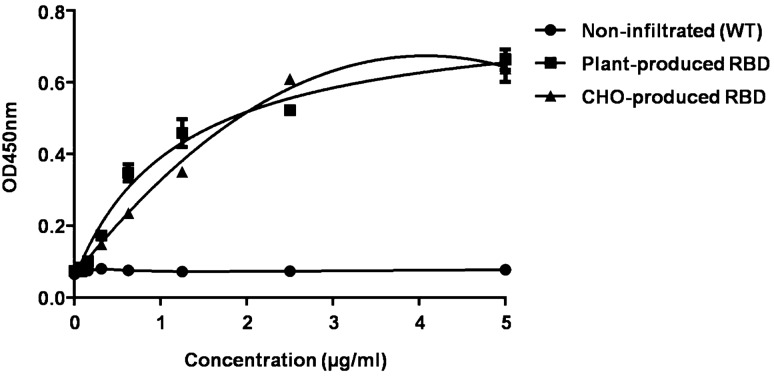
Binding of plant-produced RBD to ACE2, the receptor of SARS-CoV-2. Dilutions of plant-produced RBD and commercial CHO-produced RBD (control) were incubated on plates coated with ACE2 and detected with anti-his antibody conjugated with HRP. Non-infiltrated (WT) plant protein was used as a negative control. The data are the mean values of triplicates from each concentration.

We further tested the binding of the plant-produced RBD to ACE2, which is a SARS-CoV-2 receptor protein. Non-infiltrated wild type (WT) plant protein was used as a negative control to show that there was no cross-reactivity between ACE2 and plant proteins. The result showed that the plant-produced RBD can bind to ACE2, similar to commercial RBD protein (Fig. 4). This result indicates the authenticity and proper folding of the plant-produced RBD protein.

### Expression and purification of mAb CR3022 in *N. benthamiana*

**Figure 5:**
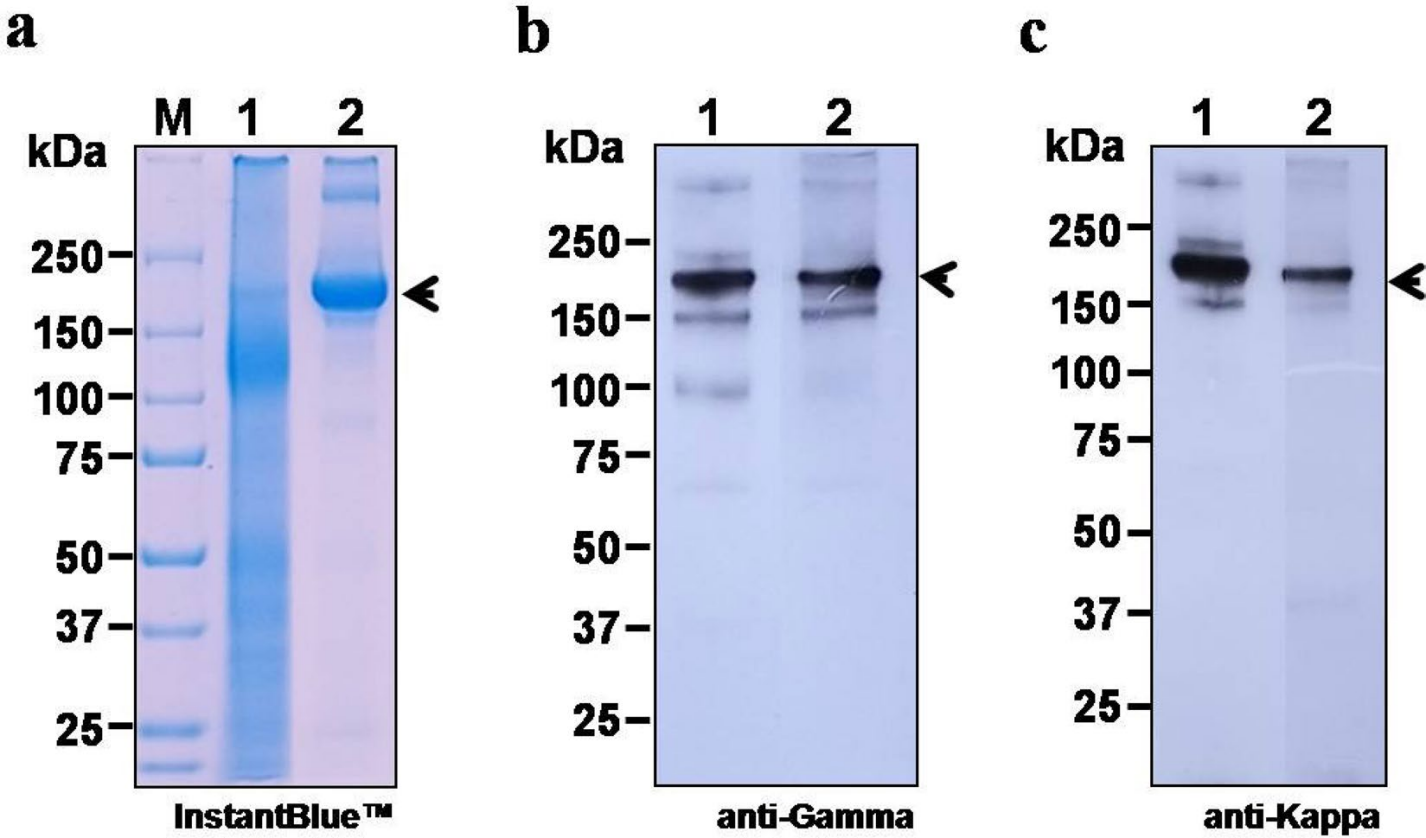
SDS-PAGE and western blot analysis of plant-produced mAb CR3022. The crude proteins were extracted from plant leaves and the antibody was purified, analyzed on SDS-PAGE gels and visualized with Instant Blue (**a**). For western blot analysis, proteins on the blot were probed with anti-human IgG gamma chain antibody conjugated with HRP (**b**) and anti-human IgG kappa chain antibody conjugated with HRP (**c**) under non-reducing conditions. Lane M: protein ladder; Lane 1: total soluble protein of *N. benthamiana* agroinfiltrated with pBY2e-CR3022 HC and LC; Lane 2: purified plant-produced mAb CR3022. Arrow head indicates full-length antibody.

Codon-optimized heavy chain (HC) and light chain (LC) expression cassettes of mAb CR3022 were cloned into pBY2e (Fig. 2). The antibody was transiently expressed in *N. benthamiana* and purified by protein A affinity chromatography. Non-reducing SDS-PAGE confirmed the tetrameric form of the fully assembled IgG at approximately 150 kDa (Fig. 5a). The assembled antibody was also confirmed by western blot using anti-human gamma (Fig. 5b) and anti-human kappa antibodies conjugated with HRP (Fig. 5c). The expression level of mAb CR3022 was estimated to be 130 μg per gram leaf fresh weight. Purified mAb CR3022 was used for further studies.

### Binding of plant-produced mAb CR3022 to RBD of SARS-CoV-2

**Figure 6:**
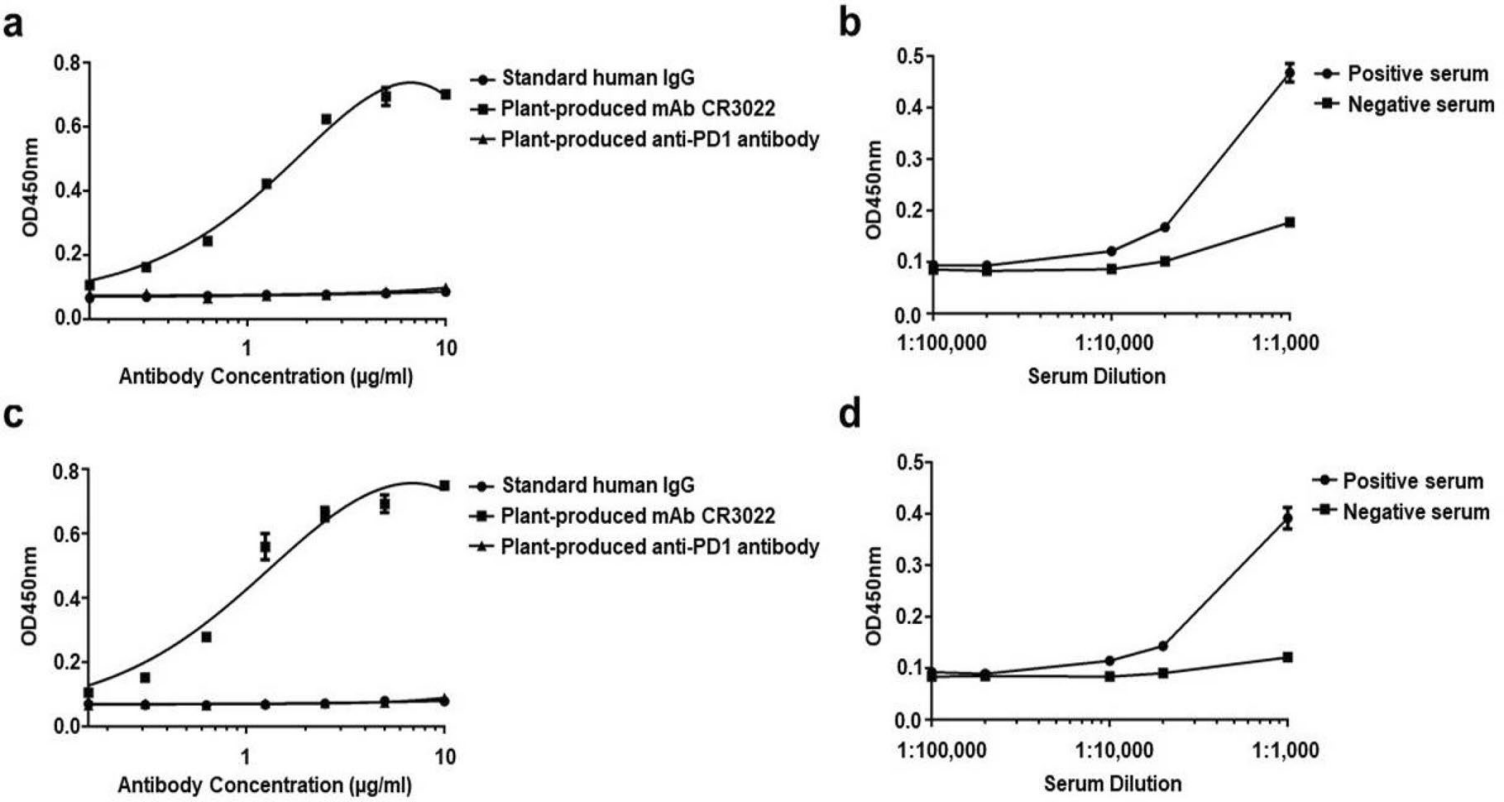
Binding of plant-produced mAb CR3022 to RBD protein. The different concentration of plant-produced mAb CR3022, standard human IgG1, and a plant-produced anti-PD1 antibody (as negative control)^28^ were incubated on plates coated either with the plant-produced SARS-CoV-2 RBD (**a**) or a commercial CHO-produced RBD (**c**) and detected with an HRP-conjugated anti-human kappa antibody. In parallel, different dilutions of positive convalescent serum collected from a COVID-19 patient and negative serum were also tested for plant-produced RBD (**b**) and CHO-produced RBD binding (**d**). The data are the mean values of triplicates from each concentration or dilution.

The binding of the plant-produced mAb CR3022 to RBD protein was examined by ELISA. Human IgG, a plant-produced anti-PD1 antibody, and negative serum were used as negative controls. Convalescent serum collected from a COVID-19 patient was used as a positive control. The plant-produced mAb CR3022 and positive serum showed specific binding to the plant-produced RBD protein (Fig. 6a,b) and commercial CHO-produced RBD protein (Fig. 6c,d) but negative controls did not bind to the RBD protein. These results confirmed that the plant-produced mAb CR3022 can specifically bind to RBD protein of SARS-CoV-2.

### Binding and neutralization activity of plant-produced mAb CR3022 against SARS-CoV-2 in vitro

**Figure 7:**
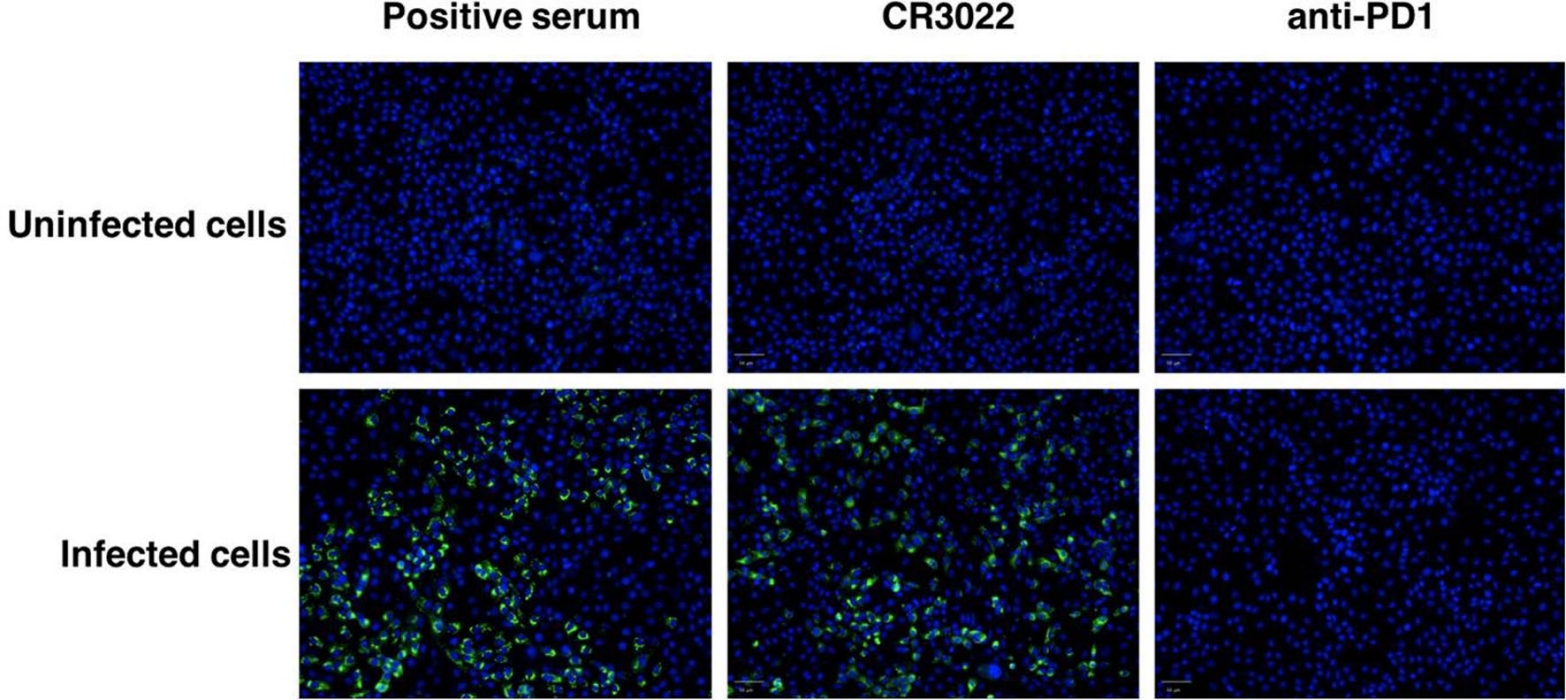
Specific binding of plant-produced mAb CR3022 to SARS-CoV-2 in infected Vero E6 cells using immunofluorescence. The plant-produced mAb CR3022, positive serum, and a plant-produced anti-PD1 antibody (as negative control) were incubated with SARS-CoV-2-infected and non-infected Vero E6 cells and signal detected with an FITC-conjugated anti-human IgG antibody (green color). Hoechst33342 was used for counterstaining (blue color). The data are the representative images of triplicate assays.

An immunofluorescence assay was performed to determine whether mAb CR3022 recognize SARS-CoV-2. SARS-CoV-2 was inoculated onto Vero cells and infected cells were incubated with a known positive serum, CR3022, and a negative control antibody^28^ before detection with an anti-human IgG conjugated with FITC. The results showed that the plant produced mAb CR3022 could bind to SARS-CoV-2 in infected cells, similar to the positive control serum (Fig. 7). To test the neutralizing activity of mAb CR3022, Vero cells infected with SARS-CoV-2 developed cytopathic effects at 3 days post infection. Positive serum had a high viral neutralization titer against SARS-CoV-2 (Table 1). In contrast, mAb CR3022 and negative serum had no neutralizing activity against SARS-CoV-2. Overall, the plant-produced CR3022 can bind to SARS-CoV-2 but did not neutralize the virus in vitro.

**Table 1:** Neutralization titer against SARS-CoV-2.

Antibody	Neutralization titer
CR3022	< 20
Positive serum	320
Negative serum	< 20

Initial concentration of the plant-produced mAb CR3022 used for the experiment was 4 mg/ml.

## Discussion

The frequent outbreaks of emerging or re-emerging infectious diseases threaten global health security, as they can have devastating health and economic impact especially in the developing world. The fear and panic over the spread of epidemic diseases can disrupt economy, travel, social activities and tourism, as well as decrease trade which in turn can affect whole societies, economies and political systems. Thus, infectious diseases create a massive burden for the global economy^29^. The recent emergence and rapid spread of the novel coronavirus SARS-CoV-2 that causes COVID-19 has attracted the attention of the whole world. Millions of infected cases have been reported, and the death toll is rising daily^30^.

The receptor binding domain (RBD) located within the spike region of SARS-CoV-2 mediates virus entry into host cells by interacting with the host receptor angiotensin converting enzyme 2 (ACE2)^31^. The new virus SARS-CoV-2 is genetically related to SARS-CoV which also utilizes the ACE2 receptor on human cells for its cell attachment and entry^32^. The RBD region located in the spike glycoprotein is essential for membrane fusion and is regarded as a major target of the host antibody response. As a result, antibodies targeting the RBD region have been extensively explored as potential coronavirus therapeutic candidates and also for the development of SARS-CoV-2 diagnostics^3,33^.

In order to meet the demand for variety of reagents required for the diagnosis and detection of COVID-19, the current study evaluated the potential of plant expression system for the production of the RBD of SARS-CoV-2 and mAb CR3022 specific to the RBD of SARS-CoV-1 and SARS-CoV-2 in order to use as a diagnostic reagent for SARS-COV-2 detection. We used a geminiviral replicon vector derived from the bean yellow dwarf virus^34,35^ for the production of both RBD and mAb CR3022. Our results showed that both the RBD of SARS-CoV-2 and mAb CR3022 could be produced rapidly in *N. benthamiana* within a time frame of less than 2 weeks after the gene construct delivery^36^. Recent developments in plant expression strategies using viral vectors and transient expression has increased protein yield, significantly reduced the upstream production cost and simplified the downstream processing of plant recombinant proteins which improves the commercial viability of the system^11,37–40^. Our results indicated that the RBD of SARS-CoV-2 was expressed in *N. benthamiana* as a soluble protein rapidly and accumulated to 8 μg/g leaf fresh weight. The mAb CR3022 accumulated at high levels at 130 μg/g leaf fresh weight within 3 days post-infiltration in *N. benthamiana* leaves, similar to earlier reports showing the accumulation of other recombinant proteins within 4 days post infiltration when using geminiviral vectors^41–44^.

The plant-produced RBD protein showed specific binding to plant-produced mAb CR3022 and the SARS-CoV-2 receptor, ACE2, which confirmed its structural integrity. Interestingly, the plant-produced mAb CR3022 could bind to SARS-CoV-2 but failed to neutralize the virus in vitro. It could be due to sequence conservation in the epitopic region of the RBD between SARS-CoV-2 and SARS-CoV, which enables cross-reactive binding of SARS-CoV neutralizing mAb CR3022 with SARS-CoV-2. However, the epitope on the SARS-CoV-2 RBD recognized by CR3022 does not overlap with the receptor ACE2 binding site. This implies that CR3022 does not compete with ACE2 for binding to the RBD^45^. Although, mAb CR3022 alone cannot neutralize SARS-CoV-2 in vitro, the synergistic potential of mAb CR3022 along with other SARS-CoV-2 specific mAbs needs to be investigated for SARS-CoV-2 neutralization.

This study provides a spotlight for the rapid production of recombinant proteins in a plant expression system, which is highly needed during an infectious disease outbreak. Moreover, a specific neutralizing antibody, if identified against SARS-CoV-2 in near future, can be produced in plant system inexpensively in a short time which could be rapidly translated into clinical trials. Earlier studies showed that plant-made antibodies developed for West Nile virus, HIV, rabies lyssavirus, dengue virus and chikungunya virus have shown potent neutralization activity in vitro which demonstrates that plants are a suitable platform for mAb production^46–50^.

Moreover, earlier reports have shown that antibodies against alphavirus, cytomegalovirus and influenza virus that did not show in vitro neutralization activity were able to confer protection in in vivo studies which highlights the importance of in vivo evaluation of non-neutralizing antibodies^45,51–54^. Additionally, this mAb could also be utilized in the development of diagnostic assays for SARS-CoV-2, and the results of this study could contribute towards the low-cost development of mAbs specific diagnostic tools for SARS-CoV-2. Altogether, our results convincingly demonstrate the practicability of using a plant expression system for the rapid production of recombinant antigens and antibodies either with diagnostic or therapeutic potential. In particular, this methodology could be suitable for use in developing economies. Furthermore, this study proved the robustness of a plant transient expression system for the production of anti-SARS-CoV mAb CR3022 with high yield which can likely improve the affordability and accessibility of mAb-based diagnosis of COVID-19 in the developing world.

In summary, we have demonstrated the rapid production of SARS-CoV-2 RBD and mAb CR3022 in *N. benthamiana.* The RBD and mAb CR3022 were purified from plant extracts. Intriguingly, the plant-produced RBD exhibited specific binding to the SARS-CoV-2 receptor ACE2. The plant-produced mAb CR3022 demonstrated binding to SARS-CoV-2 but was not able to neutralize SARS-CoV-2 in vitro. However, the plant-produced mAb CR3022 can be used as a diagnostic reagent for the effective diagnosis of COVID-19. The immunogenicity, neutralizing potential, and protective efficacy of the plant-produced RBD can be studied as a potential vaccine candidate in the future. Our study provides a proof-of-principle of utilizing a plant transient expression system for the rapid production of other similar recombinant proteins that could be either used as detection/diagnostic reagents or as biotherapeutics to tackle COVID-19 or other infectious diseases.

## Materials and methods

### Construction of expression vectors for RBD and mAb CR3022

The Institutional Review Board of Chulalongkorn University approved the present study.

The coding nucleotide sequence of the RBD region located in spike protein of SARS-CoV-2 (SARS-CoV-2 RBD) (GenBank accession number: YP_009724390.1; F318-C617) was codon-optimized for *N. benthamiana* and commercially synthesized (Genewiz, Suzhou, China). The RBD was fused with an 8XHis tag at the C-terminus and cloned into a geminiviral vector (pBY2e) by using *Xba*I and *Sac*I restriction enzymes to create pBY2e-SARS-CoV-2 RBD.

The coding gene fragments of the variable heavy chain (V_H_) and variable light chain (V_L_) regions of mAb CR3022 (GenBank accession numbers: DQ168569.1 and DQ168570.1) were codon-optimized for expression in *N. benthamiana* and commercially synthesized (Genewiz, Suzhou, China). The V_H_ and V_L_ chains were fused with human IgG1 C_H_ and C_L_ regions respectively. The resulting full length coding sequences of CR3022 HC and LC were cloned into a geminiviral vector (pBY2e) as described previously^10^ by a three fragment ligation: the backbone from pBY2e was obtained from *Xba*I-*Sac*I digestion; V_H_ and C_H_ were obtained by *Xba*I-*Nhe*I and *Nhe*I-*Sac*I digestion, respectively while V_L_ and C_L_ were obtained by *Xba*I-*Afl*II and *Afl*II-*Sac*I digestion, respectively to create the expression cassettes pBY2e-CR3022 HC and pBY2e-CR3022 LC.

### Transient expression of SARS-CoV-2-RBD and mAb CR3022 in *N. benthamiana* leaves

The expression vectors were transformed into *Agrobacterium tumefaciens* strain GV3101 by electroporation, and the resulting strains were confirmed by PCR. The PCR reaction conditions were as follows: Initial denaturation at 94 °C for 5 min; 30 cycles of 94 °C for 30 s, 54 °C for 30 s, and 72 °C for 45–90 s; and a final extension at 72 °C for 10 min. The PCR products were observed on a 1% agarose gel.

Wild type *N. benthamiana* plants were grown in a green house with 8 h dark/16 h light cycle at 25 °C for 6–8 weeks. For RBD protein expression, recombinant *Agrobacterium* containing pBY2e-SARS-CoV-2 RBD was pelleted and resuspended in infiltration buffer to an OD_600_ of 0.4 and the cell suspension was delivered into *N. benthamiana* plant leaves by agroinfiltration. Similarly, for mAb CR3022 expression, recombinant *Agrobacterium* containing pBY2e-CR3022 HC and pBY2e-CR3022 LC were pelleted and resuspended in infiltration buffer to an OD_600_ of 0.4 and mixed at a 1:1 ratio prior to vacuum infiltration and infiltrated into the plant leaves.

### Extraction and purification of recombinant RBD and mAb CR3022 from plant leaves

For the RBD protein purification from the infiltrated plants, leaves were harvested at 3 days post-infiltration and extracted with extraction buffer (5 mM imidazole, 20 mM Tris–HCl pH 8.8, 50 mM NaCl) as described previously with some modifications^55^. The crude leaf extract was obtained by homogenization and clarified by centrifugation at 15,000 *g* for 30 min at 4 °C. The crude extract was purified by Ni–NTA affinity resin (Expedeon, Cambridge, UK). Then the purity of the recombinant protein was analyzed by SDS-PAGE and the bands were visualized by Instant Blue staining (Expedeon, Cambridge, UK) and detected by western blot probed with a rabbit anti-His antibody conjugated with HRP (ab1187; Abcam, UK). The concentration of the purified RBD protein was determined by the Bradford assay.

For the purification of recombinant mAb CR3022, the agroinfiltrated leaves were harvested after 3 days and proteins extracted in extraction buffer (137 mM NaCl, 2.7 mM KCl, 4.3 mM Na_2_HPO_4_, 1.47 mM KH_2_PO_4_) at pH 7.4 using a previously developed method with some modifications^28^. The crude leaf extract was obtained by homogenization and clarified by centrifugation at 15,000 *g* for 30 min at 4 °C. The recombinant protein in the clarified plant extract was purified by using protein A resin (Expedeon, Cambridge, UK). The recombinant purified antibody was analyzed by SDS-PAGE and the bands were visualized by Instant Blue staining. For western blot analysis of mAb CR3022, the separated proteins were transferred onto nitrocellulose membranes and detected either with anti-human kappa light chain (2060–05; Southern Biotech, USA) or anti-gamma heavy chain (AP004; The Binding site, UK) antibodies conjugated with horseradish peroxidase (HRP).

### Binding of plant-produced RBD to the ACE2 receptor

The binding affinity of the plant-produced RBD to ACE2 was analyzed by ELISA. Briefly a 96-well plate (Greiner Bio-One GmbH, Germany) was coated with 2 μg/ml of commercially available ACE2 (ab273687, Abcam, UK) and incubated overnight at 4ºC. After incubation, the coating buffer was discarded and the plates were blocked with 5% skim milk in 1XPBS for 2 h at 37 °C. The plate was then washed three times with 1X PBST and incubated with the plant-produced RBD or commercial recombinant CHO-derived SARS-CoV-2 spike RBD protein (R & D Systems, USA) for 2 h at 37 °C and non-infiltrated (WT) plant protein was used as a control. Then the plates were washed three times with 1X PBST followed after which an anti 6xHis antibody (ab1187, Abcam, UK) diluted (1:1000) in 1X PBS was added to the plate which was then incubated for 1 h at 37 °C. Finally the plate was washed with 1X PBST, and the signal developed with TMB substrate (Promega, USA) and the absorbance read at 450 nm.

### Binding of plant-produced mAb CR3022 to the RBD of SARS-CoV-2

ELISA was performed as described previously to examine the binding of mAb CR3022 with RBD^26^ with some modifications. Briefly, 50 μl (2 μg/ml) of the plant-purified SARS-CoV-2 RBD or commercial recombinant CHO-derived SARS-CoV-2 spike RBD-His protein (10534-CV, R & D Systems, USA) was coated on 96-well microplates (Greiner Bio-One GmbH, Germany) and incubated at 4 °C overnight. After washing, the plates were blocked with 5% skim milk (BD, Franklin Lakes, NJ) in 1X PBS for 2 h at 37 °C. Then, the plant-produced mAb CR3022 was added in triplicate twofold serial dilutions to the plate. After 2 h incubation at 37 °C, sheep anti-human kappa light chain conjugated with HRP (The Binding Site, UK) at a dilution of 1:1000 in 1X PBS was added and samples were incubated for 1 h at 37 °C. The plate was then washed three times with PBST, and the signal developed with TMB substrate (R&D system, USA) and the absorbance read at 450 nm. Positive convalescent serum collected from a COVID-19 patient was used as positive control. The commercially available human IgG1 (ab206198; Abcam, UK), a plant produced anti-PD1 antibody^28^ and negative serum were used as negative controls.

### Virus and cells

The Institutional Review Board of Mahidol University approved the present study. Vero and Vero E6 cells were incubated at 37 °C and 5% CO_2_ in a humidified incubator. Cells were grown in DMEM medium (Gibco, NY, USA) supplemented with 10% fetal bovine serum (Gibco, NY, USA), 100 U/mL of penicillin and 0.1 mg/ml of streptomycin. A SARS-CoV-2 isolate (SARS-CoV-2/01/human/Jan2020/Thailand) isolated from a confirmed COVID-19 patient at Bamrasnaradura Infectious Diseases Institute, Nonthaburi, Thailand was grown in Vero cells. The virus stock used in the experiments had undergone one passage in Vero E6 cells. Virus titers were quantitated as TCID50/ml in confluent cells in 96-well microtiter plates and stored at − 80 °C before use. All the experiments with live SARS-CoV-2 virus were performed at a certified biosafety level 3 facility, Department of Microbiology, Faculty of Science, Mahidol University, Thailand. The experimental protocol was approved by Mahidol University and all methods were performed in accordance with the relevant guidelines and regulations.

### Binding and neutralization of plant-derived mAb CR3022 against SARS-CoV-2

Neutralizing titers were determined by a microneutralization assay. A positive convalescent serum of a COVID-19 patient was approved for use as a clinical specimen by the Faculty of Medicine Ramathibodi Hospital, Mahidol University. Informed consent was waived by the Institutional Review Board that approved the present study. The mAbs or positive serum were serially diluted twofold and incubated with 100 TCID50 of the SARS-CoV-2 virus for 1 h at 37 °C. The virus and antibodies were then added to a 96-well plate with 1 × 10^4^ Vero E6 cells/well in DMEM supplemented with 2% FBS, 100 U/ml of penicillin and 0.1 mg/ml of streptomycin in quadruplicate. Wells were observed for cytopathic effect (CPE) at 3 days post infection, and the 50% neutralization titer was determined as the mAb concentration at which at least 50% of wells revealed no CPE. Anti-SARS-CoV mAb binding was detected by immunofluorescence. Vero E6 cell monolayers in 96 wells were inoculated with 10 TCID_50_ SARS-CoV-2 and incubated for 3 days. Uninfected and infected cells were washed three times with PBS, then incubated with ice-cold 1:1 methanol/acetone fixative for 20 min at 4 °C then washed 3 times with PBST. Blocking reagent (2% bovine serum albumin, BSA) was added to the wells, and plates were incubated for 1 h at room temperature. After washing, the mAbs or the positive serum at a dilution factor 1:40 were added and the samples were incubated at 37 °C for 1 h. The antibodies were detected using a 1:1000 dilution of an anti-human IgG antibody conjugated with FITC (Santa Cruz Biotechnology, Inc.). After incubation at 37 °C for 1 h, the plate was washed three times and the DNA staining dye, Hoechst33342 was added. The plate was then subjected to automated image acquisition and analysis using Operetta (PerkinElmer). All sera were heat inactivated at 56 °C for 30 min before use.

## Supplementary information

Supplementary Figures.

## Data Availability

The datasets used and analyzed in this study are available from the corresponding author upon request.
