# Supplementary material for: Rapid production of SARS-CoV-2 receptor binding domain (RBD) and spike specific monoclonal antibody CR3022 in Nicotiana benthamiana
Source: Sci Rep. 2020 Oct 19;10:17698. doi: 10.1038/s41598-020-74904-1 (PMC7573609; doi:10.1038/s41598-020-74904-1)
Supplement: Supplementary file 1 — Supplementary Figures. [file 41598_2020_74904_MOESM1_ESM.doc]

*Supplementary information*

**Rapid Production of SARS-CoV-2 Receptor Binding Domain (RBD) and Spike Specific Monoclonal Antibody CR3022 in *Nicotiana benthamiana***

### Kaewta Rattanapisit^1, 2, §^, Balamurugan Shanmugaraj^1, 2, §^, **Suwimon Manopwisedjaroen^3^, Priyo Budi Purwono^3,4^,** Konlavat Siriwattananon^2^, Narach Khorattanakulchai^2^, Oranicha Hanittinan ^2^, Wanuttha Boonyayothin^2^, Arunee Thitithanyanont^3^, Duncan R. Smith^5^, Waranyoo Phoolcharoen^1, 2^*

^1^ Research unit for Plant-produced Pharmaceuticals, Chulalongkorn University, Bangkok, Thailand

^2^ Department of Pharmacognosy and Pharmaceutical Botany, Faculty of Pharmaceutical Sciences, Chulalongkorn University, Bangkok, Thailand.

^3^ Department of Microbiology, Faculty of Science, Mahidol University, Bangkok, Thailand

^4^ Department of Microbiology, Faculty of Medicine, Universitas Airlangga, Surabaya, Indonesia

^5^ Institute of Molecular Biosciences, Mahidol University, Salaya, Nakhon Pathom, Thailand

*****Correspondence: Waranyoo.P@chula.ac.th; Tel: 662-218-8359; Fax: 662-218-8357

^§^These authors contributed equally to this work.


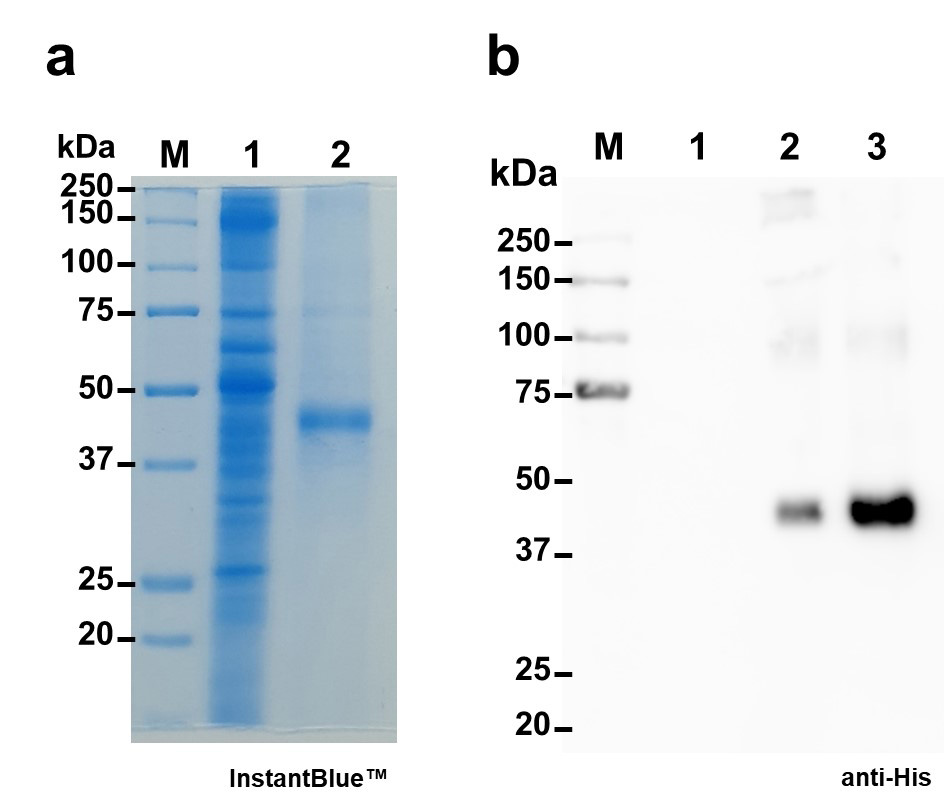
**Figure S1**

**Figure S1**: SDS-PAGE and western blot analysis of RBD protein of SARS-CoV-2 produced in *N. benthamiana*. The crude proteins were extracted from plants, RBD antigen was purified and analyzed on SDS-PAGE gels and visualized with InstantBlue™ (a). Lane M: protein ladder; Lane 1: total soluble protein of *N. benthamiana* agroinfiltrated with pBY2e-SARS-CoV-2 RBD; Lane 2: purified SARS-CoV-2 RBD. For western blot analysis, proteins on the blot were probed with rabbit anti-His conjugated with HRP (b). Lane 1: crude extract from non-infiltrated *N. benthamiana*; Lane 2: total soluble protein of *N. benthamiana* agroinfiltrated with pBY2e-SARS-CoV-2 RBD; Lane 3: purified SARS-CoV-2 RBD.


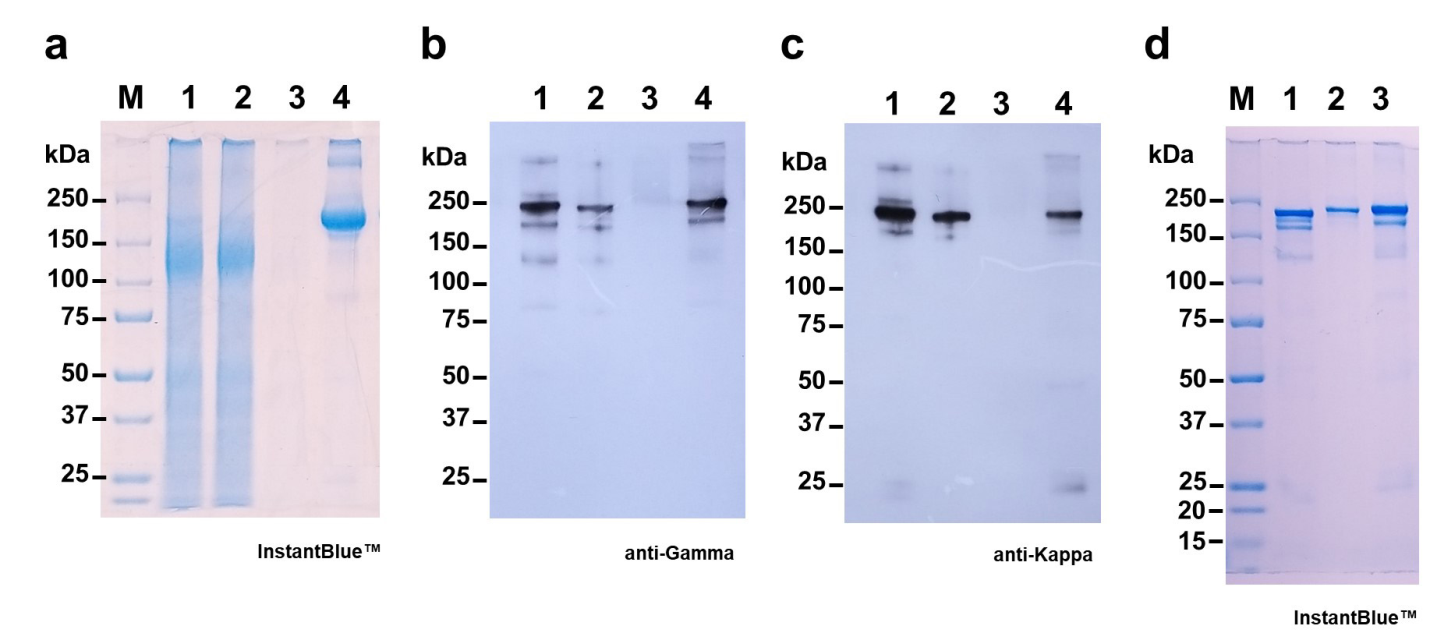
**Figure S2**

**Figure S2**: SDS-PAGE and western blot analysis of plant-produced mAb CR3022. The crude proteins were extracted from plants and the antibody was purified and analyzed on SDS-PAGE gels and visualized with InstantBlue™ (a). For western blot analysis, proteins on the blot were probed with anti-human IgG gamma chain conjugated with HRP (b) and anti-human IgG kappa chain conjugated with HRP (c) under non-reducing conditions. Lane M: protein ladder; Lane 1: total soluble protein of *N. benthamiana* agroinfiltrated with pBY2e-CR3022 HC and LC; Lane 2: flow though; Lane 3: wash; Lane 4: purified plant-produced mAb CR3022.
